# *N*-(2,3-Dimethyl­phen­yl)-4-methyl­benzene­sulfonamide

**DOI:** 10.1107/S1600536810048786

**Published:** 2010-11-27

**Authors:** P. G. Nirmala, Sabine Foro, B. Thimme Gowda, Hartmut Fuess

**Affiliations:** a Department of Chemistry, Mangalore University, Mangalagangotri 574 199, Mangalore, India; b Institute of Materials Science, Darmstadt University of Technology, Petersenstrasse 23, D-64287, Darmstadt, Germany; c Institute of Materials Science, Darmstadt University of Technology, Petersenstrasse 23, D-64287 Darmstadt, Germany

## Abstract

In the title compound, C_15_H_17_NO_2_S, the dihedral angle between the aromatic rings is 38.3 (1)°. The conformation of the N—H bond is *anti* to the methyl groups in the adjacent aromatic ring. In the crystal, N—H⋯O hydrogen bonds link the molecules into infinite chains.

## Related literature

For the preparation of the title compound, see: Shetty & Gowda (2005 ▶). For our study of the effects of substituents on the structures of *N*-(ar­yl)-aryl­sulfonamides, see: Gowda *et al.* (2009*a* ▶,*b* ▶; 2010 ▶); For related structures, see: Gelbrich *et al.* (2007 ▶); Perlovich *et al.* (2006 ▶)
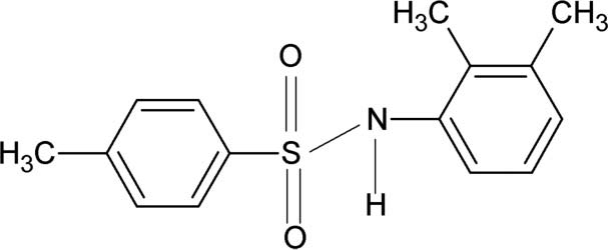


## Experimental

### 

#### Crystal data

C_15_H_17_NO_2_S
*M* *_r_* = 275.36
Monoclinic, 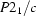

*a* = 10.771 (1) Å
*b* = 13.357 (1) Å
*c* = 9.9667 (9) Å
β = 93.256 (8)°
*V* = 1431.6 (2) Å^3^
*Z* = 4
Mo *K*α radiation
μ = 0.22 mm^−1^
*T* = 299 K
0.40 × 0.28 × 0.24 mm

#### Data collection

Oxford Diffraction Xcalibur diffractometer with a Sapphire CCD detector
Absorption correction: multi-scan (*CrysAlis RED*; Oxford Diffraction, 2009 ▶) *T*_min_ = 0.916, *T*_max_ = 0.948
5990 measured reflections
2924 independent reflections
2283 reflections with *I* > 2σ(*I*)
*R*_int_ = 0.016

#### Refinement

*R*[*F* ^2^ > 2σ(*F* ^2^)] = 0.045
*wR*(*F* ^2^) = 0.118
*S* = 1.05
2924 reflections
178 parameters
1 restraint
H atoms treated by a mixture of independent and constrained refinement
Δρ_max_ = 0.21 e Å^−3^
Δρ_min_ = −0.33 e Å^−3^

### 

Data collection: *CrysAlis CCD* (Oxford Diffraction, 2009 ▶); cell refinement: *CrysAlis RED* (Oxford Diffraction, 2009 ▶); data reduction: *CrysAlis RED*; program(s) used to solve structure: *SHELXS97* (Sheldrick, 2008 ▶); program(s) used to refine structure: *SHELXL97* (Sheldrick, 2008 ▶); molecular graphics: *PLATON* (Spek, 2009 ▶); software used to prepare material for publication: *SHELXL97*.

## Supplementary Material

Crystal structure: contains datablocks I, global. DOI: 10.1107/S1600536810048786/bq2256sup1.cif

Structure factors: contains datablocks I. DOI: 10.1107/S1600536810048786/bq2256Isup2.hkl

Additional supplementary materials:  crystallographic information; 3D view; checkCIF report

## Figures and Tables

**Table 1:** Hydrogen-bond geometry (Å, °)

*D*—H⋯*A*	*D*—H	H⋯*A*	*D*⋯*A*	*D*—H⋯*A*
N1—H1*N*⋯O1^i^	0.85 (1)	2.08 (1)	2.8999 (19)	165 (2)

Symmetry code: (i) 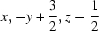.

**Fig. 1.**
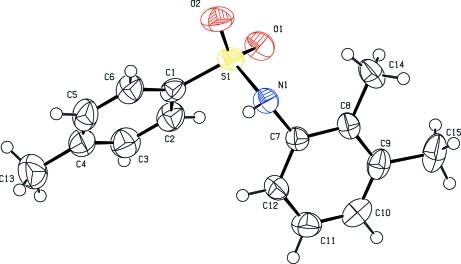
Molecular structure of (I), showing the atom labeling scheme and displacement ellipsoids are drawn at the 50% probability level.

**Fig. 2.**
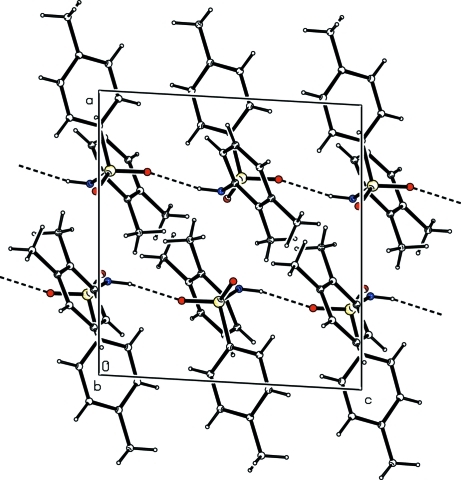
Molecular packing of (I) with hydrogen bonding shown as dashed lines.

## supplementary crystallographic information

### Comment

As part of a study of the substituent effects on the crystal structures of *N*-(aryl)-arylsulfonamides (Gowda *et al.*, 2009**a*, b*; 2010), the structure of 4-methyl-*N*-(2,3-dimethylphenyl)-benzenesulfonamide (I) has been determined (Fig. 1). The conformation of the N—H bond in (I) is *anti* to the *ortho*- and *meta*-methyl groups. The molecule is bent at the S atom with the C—SO_2_—NH—C torsion angle of 80.2 (2)°, compared to the values of -61.0 (2)° in 4-methyl-*N*-(2,5-dimethylphenyl)-benzenesulfonamide (II) (Gowda *et al.*, 2010), -51.6 (3)° in 4-Methyl-*N*-(phenyl)- benzenesulfonamide (III) (Gowda *et al.*, 2009*b*) and 71.0 (2)° in *N*-(2,3-dimethylphenyl)benzenesulfonamide (IV) (Gowda *et al.*, 2009*a*). The two benzene rings in (I) are tilted relative to each other by 38.3 (1)°, compared to the values of 49.4 (1)° in (II), 68.4 (1)° in (III) and 64.8 (1)° in (IV). The other bond parameters in (I) are similar to those observed in (II), (III), (IV) and other aryl sulfonamides (Perlovich *et al.*, 2006; Gelbrich *et al.*, 2007). The packing of molecules *via* N—H···O hydrogen bonds (Table 1) into infinite chains is shown in Fig. 2.

### Experimental

The solution of toluene (10 ml) in chloroform (40 ml) was treated dropwise with chlorosulfonic acid (25 ml) at 0 ° C. After the initial evolution of hydrogen chloride subsided, the reaction mixture was brought to room temperature and poured into crushed ice in a beaker. The chloroform layer was separated, washed with cold water and allowed to evaporate slowly. The residual 4-methylbenzenesulfonylchloride was treated with 2,3-dimethylaniline in the stoichiometric ratio and boiled for ten minutes. The reaction mixture was then cooled to room temperature and added to ice cold water (100 ml). The resultant 4-methyl-*N*-(2,3-dimethylphenyl)benzenesulfonamide was filtered under suction and washed thoroughly with cold water. It was then recrystallized to constant melting point from dilute ethanol. The purity of the compound was checked and characterized by recording its infrared and NMR spectra (Shetty & Gowda, 2005).

Rod like colourless single crystals used in X-ray diffraction studies were grown in ethanolic solution by a slow evaporation at room temperature.

### Refinement

The H atom of the NH group was located in a difference map and later restrained to the distance N—H = 0.86 (1) Å. The other H atoms were positioned with idealized geometry using a riding model with C—H = 0.93–0.96 Å. All H atoms were refined with isotropic displacement parameters (set to 1.2 times of the *U*_eq_ of the parent atom).

### Crystal data

**Table:**

C_15_H_17_NO_2_S	*F*(000) = 584
*M**_r_* = 275.36	*D*_x_ = 1.278 Mg m^−^^3^
Monoclinic, *P*2_1_/*c*	Mo *K*α radiation, λ = 0.71073 Å
Hall symbol: -P 2ybc	Cell parameters from 2134 reflections
*a* = 10.771 (1) Å	θ = 2.7–27.8°
*b* = 13.357 (1) Å	µ = 0.22 mm^−^^1^
*c* = 9.9667 (9) Å	*T* = 299 K
β = 93.256 (8)°	Rod, colourless
*V* = 1431.6 (2) Å^3^	0.40 × 0.28 × 0.24 mm
*Z* = 4	

### Data collection

**Table:**

Oxford Diffraction Xcalibur diffractometer with a Sapphire CCD detector	2924 independent reflections
Radiation source: fine-focus sealed tube	2283 reflections with *I* > 2σ(*I*)
graphite	*R*_int_ = 0.016
Rotation method data acquisition using ω and phi scans.	θ_max_ = 26.4°, θ_min_ = 3.1°
Absorption correction: multi-scan (*CrysAlis RED*; Oxford Diffraction, 2009)	*h* = −13→13
*T*_min_ = 0.916, *T*_max_ = 0.948	*k* = −16→16
5990 measured reflections	*l* = −12→12

### Refinement

**Table:**

Refinement on *F*^2^	Primary atom site location: structure-invariant direct methods
Least-squares matrix: full	Secondary atom site location: difference Fourier map
*R*[*F*^2^ > 2σ(*F*^2^)] = 0.045	Hydrogen site location: inferred from neighbouring sites
*wR*(*F*^2^) = 0.118	H atoms treated by a mixture of independent and constrained refinement
*S* = 1.05	*w* = 1/[σ^2^(*F*_o_^2^) + (0.0541*P*)^2^ + 0.5306*P*] where *P* = (*F*_o_^2^ + 2*F*_c_^2^)/3
2924 reflections	(Δ/σ)_max_ = 0.003
178 parameters	Δρ_max_ = 0.21 e Å^−^^3^
1 restraint	Δρ_min_ = −0.33 e Å^−^^3^

### Special details

**Table:**

Experimental. CrysAlis RED (Oxford Diffraction, 2009) Empirical absorption correction using spherical harmonics, implemented in SCALE3 ABSPACK scaling algorithm.
Geometry. All e.s.d.'s (except the e.s.d. in the dihedral angle between two l.s. planes) are estimated using the full covariance matrix. The cell e.s.d.'s are taken into account individually in the estimation of e.s.d.'s in distances, angles and torsion angles; correlations between e.s.d.'s in cell parameters are only used when they are defined by crystal symmetry. An approximate (isotropic) treatment of cell e.s.d.'s is used for estimating e.s.d.'s involving l.s. planes.
Refinement. Refinement of *F*^2^ against ALL reflections. The weighted *R*-factor *wR* and goodness of fit *S* are based on *F*^2^, conventional *R*-factors *R* are based on *F*, with *F* set to zero for negative *F*^2^. The threshold expression of *F*^2^ > σ(*F*^2^) is used only for calculating *R*-factors(gt) *etc*. and is not relevant to the choice of reflections for refinement. *R*-factors based on *F*^2^ are statistically about twice as large as those based on *F*, and *R*- factors based on ALL data will be even larger.

### Fractional atomic coordinates and isotropic or equivalent isotropic displacement parameters (Å^2^)

**Table:**

	*x*	*y*	*z*	*U*_iso_*/*U*_eq_	
C1	0.8751 (2)	0.83403 (14)	0.00059 (18)	0.0416 (5)	
C2	0.9745 (2)	0.79149 (16)	0.0754 (2)	0.0541 (6)	
H2	0.9603	0.7533	0.1511	0.065*	
C3	1.0950 (2)	0.80616 (18)	0.0367 (3)	0.0618 (6)	
H3	1.1610	0.7773	0.0870	0.074*	
C4	1.1192 (2)	0.86297 (18)	−0.0755 (2)	0.0597 (6)	
C5	1.0186 (3)	0.90574 (18)	−0.1467 (2)	0.0615 (6)	
H5	1.0330	0.9452	−0.2212	0.074*	
C6	0.8977 (2)	0.89196 (16)	−0.1112 (2)	0.0532 (5)	
H6	0.8320	0.9212	−0.1616	0.064*	
C7	0.70153 (17)	0.61341 (14)	0.01859 (17)	0.0370 (4)	
C8	0.62354 (17)	0.56874 (15)	0.10963 (17)	0.0388 (4)	
C9	0.6555 (2)	0.47214 (16)	0.15710 (19)	0.0474 (5)	
C10	0.7613 (2)	0.42509 (17)	0.1120 (2)	0.0566 (6)	
H10	0.7829	0.3619	0.1450	0.068*	
C11	0.8345 (2)	0.46986 (18)	0.0199 (2)	0.0574 (6)	
H11	0.9033	0.4363	−0.0104	0.069*	
C12	0.80522 (18)	0.56454 (16)	−0.0271 (2)	0.0468 (5)	
H12	0.8543	0.5955	−0.0887	0.056*	
C13	1.2511 (3)	0.8780 (2)	−0.1188 (3)	0.0863 (9)	
H13A	1.3053	0.8298	−0.0743	0.104*	
H13B	1.2788	0.9443	−0.0951	0.104*	
H13C	1.2526	0.8693	−0.2143	0.104*	
C14	0.50770 (19)	0.62004 (18)	0.1532 (2)	0.0541 (6)	
H14A	0.4944	0.6807	0.1027	0.065*	
H14B	0.5174	0.6356	0.2473	0.065*	
H14C	0.4375	0.5764	0.1374	0.065*	
C15	0.5750 (3)	0.4194 (2)	0.2541 (2)	0.0715 (8)	
H15A	0.4917	0.4140	0.2150	0.086*	
H15B	0.5744	0.4571	0.3361	0.086*	
H15C	0.6077	0.3537	0.2728	0.086*	
N1	0.66972 (16)	0.71156 (12)	−0.03365 (15)	0.0418 (4)	
H1N	0.674 (2)	0.7188 (16)	−0.1175 (10)	0.050*	
O1	0.72272 (15)	0.79141 (12)	0.18435 (13)	0.0561 (4)	
O2	0.64468 (16)	0.89283 (12)	−0.01147 (17)	0.0629 (4)	
S1	0.71972 (5)	0.81273 (4)	0.04301 (5)	0.04238 (17)	

### Atomic displacement parameters (Å^2^)

**Table:**

	*U*^11^	*U*^22^	*U*^33^	*U*^12^	*U*^13^	*U*^23^
C1	0.0596 (12)	0.0339 (10)	0.0315 (9)	−0.0002 (9)	0.0038 (8)	−0.0015 (8)
C2	0.0682 (15)	0.0489 (13)	0.0451 (12)	0.0008 (11)	0.0017 (11)	0.0089 (10)
C3	0.0572 (14)	0.0599 (15)	0.0678 (16)	−0.0013 (12)	−0.0016 (12)	−0.0013 (12)
C4	0.0666 (15)	0.0534 (14)	0.0606 (14)	−0.0176 (12)	0.0150 (12)	−0.0203 (12)
C5	0.0842 (18)	0.0578 (14)	0.0438 (12)	−0.0181 (13)	0.0151 (12)	0.0006 (11)
C6	0.0710 (15)	0.0511 (13)	0.0377 (11)	−0.0025 (11)	0.0035 (10)	0.0070 (9)
C7	0.0402 (10)	0.0413 (10)	0.0295 (9)	−0.0032 (8)	0.0026 (7)	−0.0030 (8)
C8	0.0385 (10)	0.0495 (12)	0.0284 (9)	−0.0088 (9)	0.0018 (7)	−0.0042 (8)
C9	0.0575 (13)	0.0475 (12)	0.0365 (10)	−0.0180 (10)	−0.0030 (9)	−0.0004 (9)
C10	0.0684 (15)	0.0395 (12)	0.0606 (14)	−0.0015 (11)	−0.0082 (12)	0.0003 (10)
C11	0.0512 (13)	0.0514 (13)	0.0701 (15)	0.0085 (11)	0.0066 (11)	−0.0062 (11)
C12	0.0450 (11)	0.0494 (12)	0.0472 (11)	−0.0024 (9)	0.0133 (9)	−0.0052 (9)
C13	0.0745 (18)	0.086 (2)	0.101 (2)	−0.0270 (16)	0.0265 (16)	−0.0226 (17)
C14	0.0445 (11)	0.0740 (16)	0.0450 (12)	−0.0075 (11)	0.0133 (9)	−0.0088 (11)
C15	0.096 (2)	0.0647 (16)	0.0538 (14)	−0.0357 (15)	0.0068 (13)	0.0085 (12)
N1	0.0550 (10)	0.0458 (9)	0.0248 (7)	0.0013 (8)	0.0052 (7)	0.0017 (7)
O1	0.0795 (11)	0.0646 (10)	0.0252 (7)	−0.0021 (8)	0.0122 (7)	−0.0056 (6)
O2	0.0750 (11)	0.0496 (9)	0.0645 (10)	0.0206 (8)	0.0076 (8)	0.0036 (8)
S1	0.0589 (3)	0.0400 (3)	0.0290 (2)	0.0067 (2)	0.0089 (2)	−0.00128 (19)

### Geometric parameters (Å, °)

**Table:**

C1—C6	1.389 (3)	C10—C11	1.381 (3)
C1—C2	1.391 (3)	C10—H10	0.9300
C1—S1	1.773 (2)	C11—C12	1.379 (3)
C2—C3	1.389 (3)	C11—H11	0.9300
C2—H2	0.9300	C12—H12	0.9300
C3—C4	1.388 (3)	C13—H13A	0.9600
C3—H3	0.9300	C13—H13B	0.9600
C4—C5	1.385 (4)	C13—H13C	0.9600
C4—C13	1.522 (3)	C14—H14A	0.9600
C5—C6	1.381 (3)	C14—H14B	0.9600
C5—H5	0.9300	C14—H14C	0.9600
C6—H6	0.9300	C15—H15A	0.9600
C7—C12	1.392 (3)	C15—H15B	0.9600
C7—C8	1.404 (2)	C15—H15C	0.9600
C7—N1	1.445 (2)	N1—S1	1.6288 (17)
C8—C9	1.410 (3)	N1—H1N	0.845 (9)
C8—C14	1.509 (3)	O1—S1	1.4357 (14)
C9—C10	1.398 (3)	O2—S1	1.4294 (16)
C9—C15	1.509 (3)		
			
C6—C1—C2	119.6 (2)	C10—C11—H11	120.2
C6—C1—S1	119.42 (17)	C11—C12—C7	119.44 (19)
C2—C1—S1	121.00 (15)	C11—C12—H12	120.3
C3—C2—C1	119.8 (2)	C7—C12—H12	120.3
C3—C2—H2	120.1	C4—C13—H13A	109.5
C1—C2—H2	120.1	C4—C13—H13B	109.5
C4—C3—C2	121.5 (2)	H13A—C13—H13B	109.5
C4—C3—H3	119.3	C4—C13—H13C	109.5
C2—C3—H3	119.3	H13A—C13—H13C	109.5
C5—C4—C3	117.5 (2)	H13B—C13—H13C	109.5
C5—C4—C13	121.1 (2)	C8—C14—H14A	109.5
C3—C4—C13	121.4 (3)	C8—C14—H14B	109.5
C6—C5—C4	122.4 (2)	H14A—C14—H14B	109.5
C6—C5—H5	118.8	C8—C14—H14C	109.5
C4—C5—H5	118.8	H14A—C14—H14C	109.5
C5—C6—C1	119.3 (2)	H14B—C14—H14C	109.5
C5—C6—H6	120.3	C9—C15—H15A	109.5
C1—C6—H6	120.3	C9—C15—H15B	109.5
C12—C7—C8	122.17 (19)	H15A—C15—H15B	109.5
C12—C7—N1	119.07 (16)	C9—C15—H15C	109.5
C8—C7—N1	118.70 (17)	H15A—C15—H15C	109.5
C7—C8—C9	117.52 (18)	H15B—C15—H15C	109.5
C7—C8—C14	121.86 (19)	C7—N1—S1	121.23 (13)
C9—C8—C14	120.59 (18)	C7—N1—H1N	115.7 (15)
C10—C9—C8	119.44 (18)	S1—N1—H1N	109.4 (15)
C10—C9—C15	120.3 (2)	O2—S1—O1	120.12 (9)
C8—C9—C15	120.2 (2)	O2—S1—N1	106.14 (10)
C11—C10—C9	121.8 (2)	O1—S1—N1	106.51 (9)
C11—C10—H10	119.1	O2—S1—C1	108.09 (10)
C9—C10—H10	119.1	O1—S1—C1	107.27 (10)
C12—C11—C10	119.6 (2)	N1—S1—C1	108.23 (9)
C12—C11—H11	120.2		
			
C6—C1—C2—C3	−0.9 (3)	C8—C9—C10—C11	−1.3 (3)
S1—C1—C2—C3	177.42 (17)	C15—C9—C10—C11	177.8 (2)
C1—C2—C3—C4	0.2 (4)	C9—C10—C11—C12	1.7 (4)
C2—C3—C4—C5	0.8 (3)	C10—C11—C12—C7	−0.4 (3)
C2—C3—C4—C13	−179.3 (2)	C8—C7—C12—C11	−1.2 (3)
C3—C4—C5—C6	−1.2 (3)	N1—C7—C12—C11	−178.47 (18)
C13—C4—C5—C6	178.8 (2)	C12—C7—N1—S1	−93.10 (19)
C4—C5—C6—C1	0.6 (3)	C8—C7—N1—S1	89.58 (19)
C2—C1—C6—C5	0.5 (3)	C7—N1—S1—O2	−163.99 (14)
S1—C1—C6—C5	−177.87 (16)	C7—N1—S1—O1	−34.88 (17)
C12—C7—C8—C9	1.6 (3)	C7—N1—S1—C1	80.18 (15)
N1—C7—C8—C9	178.83 (16)	C6—C1—S1—O2	−24.52 (19)
C12—C7—C8—C14	−176.82 (18)	C2—C1—S1—O2	157.18 (17)
N1—C7—C8—C14	0.4 (3)	C6—C1—S1—O1	−155.40 (16)
C7—C8—C9—C10	−0.3 (3)	C2—C1—S1—O1	26.30 (19)
C14—C8—C9—C10	178.10 (19)	C6—C1—S1—N1	90.03 (18)
C7—C8—C9—C15	−179.40 (18)	C2—C1—S1—N1	−88.26 (18)
C14—C8—C9—C15	−1.0 (3)		

### Hydrogen-bond geometry (Å, °)

**Table:**

*D*—H···*A*	*D*—H	H···*A*	*D*···*A*	*D*—H···*A*
N1—H1N···O1^i^	0.85 (1)	2.08 (1)	2.8999 (19)	165 (2)

Symmetry codes: (i) *x*, −*y*+3/2, *z*−1/2.
